# The effect of bed-to-nurse ratio on hospital mortality of critically ill children on mechanical ventilation: a nationwide population-based study

**DOI:** 10.1186/s13613-020-00780-7

**Published:** 2020-11-30

**Authors:** Minyoung Jung, Hyejeong Park, Danbee Kang, Esther Park, Kyeongman Jeon, Chi Ryang Chung, Jeong Hoon Yang, Gee Young Suh, Eliseo Guallar, Juhee Cho, Joongbum Cho

**Affiliations:** 1 Department of Pediatrics, Kosin University Gospel Hospital, Kosin University School of Medicine, Busan, Republic of Korea; 2 Department of Critical Care Medicine, Samsung Medical Center, Sungkyunkwan University School of Medicine, 81 Irwon-ro, Gangnam-gu, Seoul, 06351 Republic of Korea; 3 Center for Clinical Epidemiology, Samsung Medical Center, Seoul, Republic of Korea; 4 Department of Clinical Research Design & Evaluation, SAIHST, Sungkyunkwan University, Seoul, South Korea; 5 Departments of Epidemiology and Department of Medicine, Welch Center for Prevention, Epidemiology, and Clinical Research, Johns Hopkins Bloomberg School of Public Health, Baltimore, MD USA

**Keywords:** Mechanical ventilation, Nurse staffing, Critical care, Critical care outcomes, Pediatric intensive care units, Quality of care, Mortality, Epidemiology, National Health Insurance

## Abstract

**Background:**

Despite the high workload of mechanical ventilation, there has been a lack of studies on the association between nurse workforce and mortality in mechanically ventilated patients. We evaluated the association of the bed-to-nurse ratio with mortality in ventilated pediatric patients admitted to an intensive care unit (ICU).

**Methods:**

We conducted a nationwide retrospective analysis by using the Korean National Health Insurance database, which categorizes the bed-to-nurse ratio into 9 grades according to the number of beds divided by the number of full-time equivalent registered nurses in a unit. Patients of ages between 28 days and 18 years were enrolled. Multiple admissions and transfers from other hospitals were excluded. We evaluated the odds ratios (ORs) of in-hospital mortality using 4 groups (Grade 1: bed-to-nurse < 0.50, Grade 2: < 0.63, Grade 3: < 0.77, Grade 4 or above > 0.77) with adjustment of patient factors, hospital factors, and treatment requirements.

**Results:**

Of the 27,849 patients admitted to ICU, 11,628 (41.8%) were on mechanical ventilation. The overall in-hospital mortality rates in Grade 1, Grade 2, Grade 3, and Grade 4 or above group were 4.5%, 6.8%, 6.9%, and 4.7%, respectively. The adjusted ORs (95% CI) for in-hospital mortality of mechanically ventilated patients in the Grade 2, Grade 3, and Grade 4 or above compared to those in Grade 1 were 2.73 (95% CI 1.51–4.95), 4.02 (95% CI 2.23–7.26), and 7.83 (4.07–15.07), respectively. However, for patients without mechanical ventilation, the adjusted ORs of in-hospital mortality were not statistically significant.

**Conclusion:**

In mechanically ventilated patients, the adjusted mortality rate increased significantly, as the bed-to-nurse ratio of the ICU increased. Policies that limit the number of ventilated patients per nurse should be considered.

*Trial registration* retrospectively registered

## Background

The nurse staffing ratio is associated with the quality of care and safety of a hospital [1–4]. In an intensive care unit (ICU), unplanned extubation, central line-associated bloodstream infection, and ventilator-associated pneumonia are associated with a small number of nurses, although the bed-to-nurse ratio is smaller in the ICU than in the general ward [5–7]. Organizations of critical care nursing professionals suggested minimum standards on nurse staffing ratios [8, 9]. The standards specified that the number of patients assigned to one nurse should be smaller in mechanically ventilated patients than in other patients in an ICU.

Mechanical ventilation is a cause of high nursing workload in ICU [10, 11]. A high workload is associated with patient mortality [12]. However, there are high-level ICUs, even in a developed country, which do not meet the suggested standards [13]. The global shortage of registered nurses might compromise the compliance of nursing standards [14]. In addition, there is a lack of studies on the association between mortality and nurse staffing ratio in ventilated patients [15]. Results of studies on the association between nurse staffing ratio and mortality were inconsistent [16, 17].

Therefore, we evaluated the association between bed-to-nurse ratio and mortality rate according to the use of mechanical ventilation in pediatric patients in an ICU using a national database.

## Methods

### Study population

We conducted a retrospective cohort analysis of the Health Insurance Review and Assessment (HIRA) database from the Korean Ministry of Health [18, 19]. Korean National Health Insurance Service (NHIS) and Medical Aid Program (MAP) cover all Korean citizens by law. All hospitals have to claim for reimbursement of ICU treatment to Korean NHIS and MAP, and HIRA reviews the claims for payment. HIRA database included nearly all ICU admissions in Korea except administrative mistakes.

The ICU admissions were defined using the claim codes, which are mandatory for ICU management of in-hospital stays (codes AJ100-AJ590900) in all Korean hospitals. All ICU stays during the same hospitalization were considered as a single ICU admission. Hospital stays separated by < 2 days were considered as the same hospital admission.

The study population consisted of all patients < 18 years of age with at least one ICU admission (regardless of ICU types) covered by the Korean National Health System between August 30, 2009, and September 30, 2014 (*n* = 130,721 infants, children or adolescents). We excluded patients with multiple admissions during the study period (*n* = 63,156) to exclude the effect of multiple admission and transfer on the mortality rate. Since we aimed to evaluate the effects of hospitals according to nurse staffing ratio, we additionally excluded participants who were transferred within one day after discharge (*n* = 2,100). We also excluded patients who were neonates (< 28 days), admitted to an NICU (codes AJ101, AJ111, AJ121, AJ141, AJ201, AJ211, AJ221, AJ241, AJ301, AJ311, AJ321, and AJ341; *n* = 37,475) since NICU has different bed-to-nurse grade system. On the other hand, we included all children in adult ICU, since adult ICU shares the same bed-to-nurse grade system. We excluded patients admitted with a primary diagnosis code of Z00-Z99 (factors influencing health status and contact with health services; *n* = 141). The final sample was 27,849 patients (Additional file 1: Fig. S1).

The sampling procedures and representativeness of the pediatric ICU cohort have been described elsewhere [19]. Our study was reviewed by the Institutional Review Board (IRB) of Samsung Medical Center (IRB protocol 2015-11-17), and informed consent was exempted because of the use of previously collected de-identified administrative data.

### Measurement

We categorized bed-to-nurse grades according to the Korean National Health Insurance (NHI) codes of ICU admission (AJ11–AJ19, AJ21–AJ29, AJ31–AJ39). These codes were designed to reimburse hospitals for ICU care service costs according to the bed-to-nurse ratio. The Korean NHI defined the bed-to-nurse ratio as the number of beds divided by the number of full-time equivalent registered nurses in a unit. There were 9 categories of codes: Grade 1 (bed-to-nurse < 0.50, the best), Grade 2 (bed-to-nurse < 0.63), Grade 3 (bed-to-nurse < 0.77), Grade 4 (bed-to-nurse < 0.88), Grade 5 (bed-to-nurse < 1.00), Grade 6 (bed-to-nurse < 1.25), Grade 7 (bed-to-nurse < 1.50), Grade 8 (bed-to-nurse < 2.0), and Grade 9 (bed-to-nurse ≥ 2.0, the worst). When we convert the bed-to-nurse ratio to the estimated number of patients cared for by an acting nurse at a particular time, less than 2.1 patients were cared for by a nurse in Grade 1 ICU, < 2.7 patients in Grade 2 ICU, < 3.3 patients in Grade 3 ICU, and < 3.7 patients in Grade 4 ICU using the assumption of 2040 h of annual work per nurse [20, 21]. In this study, we grouped Grade 4 or above in one, because few patients were admitted to ICUs above Grade 4, and they were not appropriate for ICU categorization.

Information on interventions, demographics, and hospital characteristics was based on claim codes. Primary diagnosis was defined as the condition primarily responsible for the patient’s need for treatment or investigation using Korean Classification of Disease, sixth edition, which is the modified version of the International Classification of Disease, 10th revision adapted for use in the Korean health system [22]. Interventions for critical care included the use of mechanical ventilation for more than 3 h (Korean NHI procedure codes M5857, M5858, and M5860), extracorporeal membrane oxygenation (O1901–O1904), continuous renal replacement therapy (O7051–O7054), intermittent hemodialysis (HD; O7020), peritoneal dialysis (PD; O7062), and cardiopulmonary resuscitation (M5871, M5873, M5874, M5875, M5876, and M5877). We identified the use of vasopressor drugs such as dobutamine [Anatomic Therapeutic Chemical (ATC) codes: C01CA07], dopamine (ATC codes: C01CA04), epinephrine (ATC codes: C01CA24), and norepinephrine (ATC codes: C01CA03) for more than 2 days using Korean drug and anatomical therapeutic chemical codes [23]. We obtained information on hospital characteristics from the HIRA Medical Care Institution Database, which included information regarding type of institution, location, number of beds, facilities, and physicians. Hospitals were classified according to capacity based on number of hospital beds and number of specialties as defined by the Korean Health Law, as described in a previous study [19]. In addition, we included ICU admission volume as a hospital factor by calculating the average annual pediatric admissions to the ICU for 5 years in each hospital.

### Statistical analysis

We conducted a descriptive analysis of patient characteristics across ICU groups. Mean with standard deviation or median with interquartile range were used to describe the distribution of continuous variables. Chi-square and Student’s *t*-tests were used to compare categorical and continuous variables, respectively.

The outcome of the study was all-cause mortality. We calculated ORs with 95% confidence interval (CI) for in-hospital mortality using logistic regression. We used two models with increasing degrees of adjustment to account for potential confounding factors. Model 1 was adjusted for age, sex, and primary diagnosis as patient factors. Model 2 was adjusted for hospital factors and interventions in addition to patient factors. Since ICU admission volume and region had multicollinearity, we excluded the variables from the model.

In addition, we explored the association of bed-to-nurse ratio with in-hospital mortality in pre-specified clinically relevant subgroups defined by the use of mechanical ventilation (yes vs. no), type of hospital (tertiary vs. general or hospital), and admission department (medical vs. surgical).

All analyses were performed using SAS enterprise guide 6.1 (SAS Institute, Cary, NC, USA). Two-sided significance testing was used throughout, with a *p*-value < 0.05 considered statistically significant.

## Results

Between August 2009 and September 2014, a total of 27,849 patients were admitted to an ICU. The median (interquartile range) age was 6 (1–14) years, and 58.0% were male (Table 1). The most frequent primary diagnosis was congenital anomaly (28.6%); injury (17.3%) and neoplasms (11.3%) were the next common. Mechanical ventilation was applied to 41.8% of patients, and 20.3% of patients required vasopressors.

**Table 1:** Characteristics of patients admitted to ICU according to ICU grade in Korea, August 2009 to September 2014

Characteristics	ICU bed-to-nurse grade^b^				*p*-value
	Grade 1	Grade 2	Grade 3	Grade 4 or above	
	(*n* = 11,691)	(*n* = 5095)	(*n* = 6797)	(*n* = 4266)	
Age, years	6.0 (5.5)	7.4 (6.1)	8.0 (6.1)	10.5 (5.9)	< 0.01
Age, year, median (IQR)	4 (0.9, 11)	6 (0.9, 14)	7 (1, 14)	13 (5, 16)	< 0.01
Sex, male	6323 (54.1)	3024 (59.4)	4143 (61.0)	2663 (62.4)	< 0.01
Type of hospital					< 0.01
Tertiary hospital	10,763 (92.1)	3342 (65.6)	4134 (60.8)	338 (7.9)	
General hospital	928 (7.9)	1752 (34.4)	2650 (39.0)	3700 (86.7)	
Hospital	0 (0.0)	1 (0.0)	13 (0.2)	228 (5.3)	
ICU admission volume^a^					< 0.01
> 600 per year	2962 (25.3)	0 (0.0)	0 (0.0)	0 (0.0)	
100–600 per year	8228 (70.4)	1948 (38.2)	1520 (22.4)	28 (0.7)	
< 100 per year	501 (4.3)	3147 (61.8)	5277 (77.6)	4238 (99.3)	
Region					< 0.01
Capital	10,339 (88.4)	1550 (30.4)	877 (12.9)	750 (17.6)	
Metropolitan	10 (0.1)	1471 (28.9)	3270 (48.1)	596 (14.0)	
Province	1342 (11.5)	2074 (40.7)	2650 (39.0)	2920 (68.4)	
Admission department					< 0.01
Surgical	7773 (66.5)	3011 (59.1)	3099 (45.6)	2162 (50.7)	
Medical	3918 (33.5)	2084 (40.9)	3698 (54.4)	2104 (49.3)	
Primary diagnosis					< 0.01
Congenital anomaly	5268 (45.1)	1724 (33.8)	857 (12.6)	117 (2.7)	
Injury	314 (2.7)	812 (15.9)	1730 (25.5)	1969 (46.2)	
Neoplasms	2164 (18.5)	365 (7.2)	405 (6.0)	220 (5.2)	
Respiratory	560 (4.8)	479 (9.4)	1186 (17.4)	642 (15.0)	
Neurologic disease	1024 (8.8)	496 (9.7)	671 (9.9)	330 (7.7)	
Circulatory disease	1107 (9.5)	399 (7.8)	517 (7.6)	156 (3.7)	
Gastrointestinal disease	287 (2.5)	154 (3.0)	282 (4.1)	255 (6.0)	
Not elsewhere classified	80 (0.7)	105 (2.1)	360 (5.3)	148 (3.5)	
Infectious disease	131 (1.1)	130 (2.6)	237 (3.5)	97 (2.3)	
Others	756 (6.5)	431 (8.5)	552 (8.1)	332 (7.8)	
Interventions for critical care					
Mechanical ventilation	6610 (56.5)	2582 (50.7)	1920 (28.2)	516 (12.1)	< 0.01
Vasopressors	2696 (23.1)	1263 (24.8)	977 (14.4)	315 (7.4)	< 0.01
ECMO	142 (1.2)	53 (1.0)	58 (0.9)	13 (0.3)	< 0.01
Hemodialysis	504 (4.3)	110 (2.2)	105 (1.5)	40 (0.9)	< 0.01
CPR	382 (3.3)	298 (5.8)	404 (5.9)	161 (3.8)	< 0.01

Values are median (interquartile range) or *n* (%)

*IQR* interquartile range, *ICU* intensive care unit, *ECMO* extracorporeal membrane oxygenation, *CPR* cardiopulmonary resuscitation

^a^ICU admission volume was calculated as the average annual pediatric admissions to the ICU for 5 years in each hospital

^b^ICU bed-to-nurse grade was defined as bed-to-nurse ratio (Grade 1: < 0.5, Grade 2: < 0.63, Grade 3: < 0.77, and Grade 4 or above: > 0.77)

Among patients, 11,691 (42.0%) were in Grade 1, 5095 (18.3%) in Grade 2, 6797 (24.4%) in Grade 3, and 4266 (15.3%) in Grade 4 or above group. Compared with patients in ICU Grade 4 or above, patients in ICU Grade 1 were more likely to be younger (median 4 vs. 13, *p* < 0.01) and to require mechanical ventilation (56.5% vs. 12.1%; *p* < 0.01), vasopressor drugs (23.1% vs. 7.4%, *p* < 0.01), or hemodialysis (4.3% vs. 0.9%, *p* < 0.01). The most common primary diagnosis was injury (46.2%) in Grade 4 or above and congenital anomalies (45%) in Grade 1 (Table 1).

**Fig. 1:**
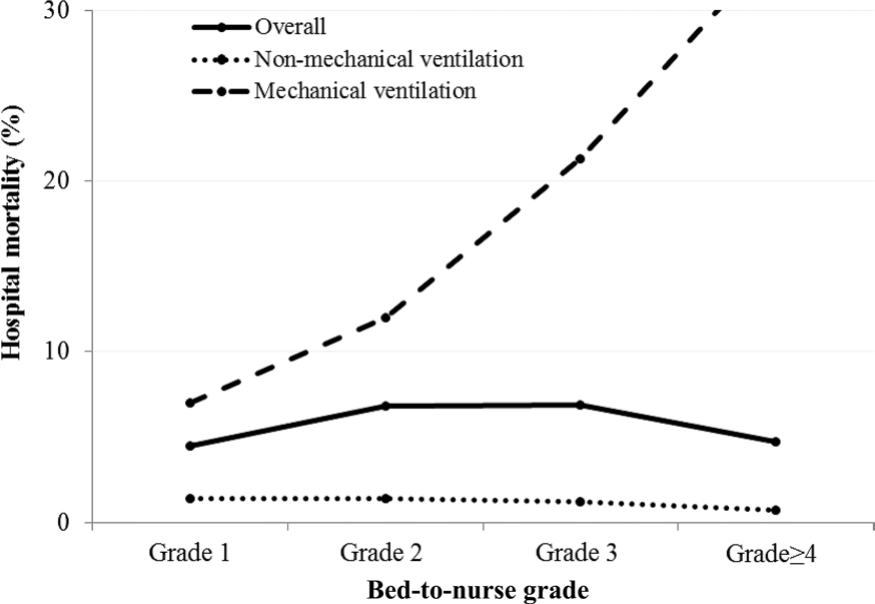
Unadjusted hospital mortality according to ICU bed-to-nurse grade

Among 27,849 patients, 1543 (5.5%) died in hospital. The in-hospital mortality rates in Grade 1, Grade 2, Grade 3, and Grade 4 or above group were 4.5%, 6.8%, 6.9%, and 4.7%, respectively (Fig. 1). The fully adjusted ORs (model 2) for in-hospital mortality for patients in ICU Grade 2, ICU Grade 3, and ICU Grade 4 or above compared with those in ICU Grade 1 were 2.49 (95% CI 1.46–4.24), 3.61 (95% CI 2.13–6.14), and 4.74 (2.65–8.46), respectively (Table 2). Among patients on mechanical ventilation (*n* = 11,682), the adjusted ORs (95% CI) for in-hospital mortality of patients in Grade 2, Grade 3, Grade 4 or above compared to patients in Grade 1 were 2.73 (95% CI 1.51–4.95), 4.02 (95% CI 2.23–7.26), and 7.83 (4.07–15.07), respectively. However, for patients without mechanical ventilation, the adjusted ORs of in-hospital mortality did not increase with statistical significance according to the increase of ICU grade.

**Table 2:** Odds ratio (95% CI) for in-hospital mortality according to ICU grade and use of mechanical ventilator in South Korea, 2009 to 2014

Bed-to-nurse grade	No. of patients	No. of deaths (%)	Model 1 OR (95% CI)	Model 2 OR (95% CI)
Overall				
Grade 1	11,691	531 (4.5)	Reference	Reference
Grade 2	5095	345 (6.8)	1.71 (1.13–2.58)	2.49 (1.46–4.24)
Grade 3	6797	468 (6.9)	1.83 (1.21–2.76)	3.61 (2.13–6.14)
Grade 4 or above	4266	199 (4.7)	1.27 (0.83–1.95)	4.74 (2.65–8.46)
*p*-value			0.003	< 0.01
No-mechanical ventilation				
Grade 1	5081	69 (1.4)	Reference	Reference
Grade 2	2513	34 (1.4)	1.36 (0.69–2.66)	1.23 (0.63–2.39)
Grade 3	4877	59 (1.2)	1.68 (0.90–3.14)	1.51 (0.80–2.86)
Grade 4 or above	3750	25 (0.7)	1.27 (0.63–2.56)	0.99 (0.45–2.14)
*p*-value			0.395	0.359
Mechanical ventilation				
Grade 1	6610	462 (7.0)	Reference	Reference
Grade 2	2582	311 (12.0)	2.00 (1.24–3.24)	2.73 (1.51–4.95)
Grade 3	1920	409 (21.3)	2.65 (1.65–4.25)	4.02 (2.23–7.26)
Grade 4 or above	516	174 (33.7)	4.15 (2.50–6.89)	7.83 (4.07–15.07)
*p*-value			< 0.01	< 0.01

Model 1 adjusted for age, sex, and primary diagnosis

Model 2 further adjusted for medical/surgical admission, hospital type, interventions for critical care (vasopressor drugs, extracorporeal membrane oxygenation, and hemodialysis)

*CI* confidence interval, *OR* odds ratio, *ICU* intensive care unit

**Fig. 2:**
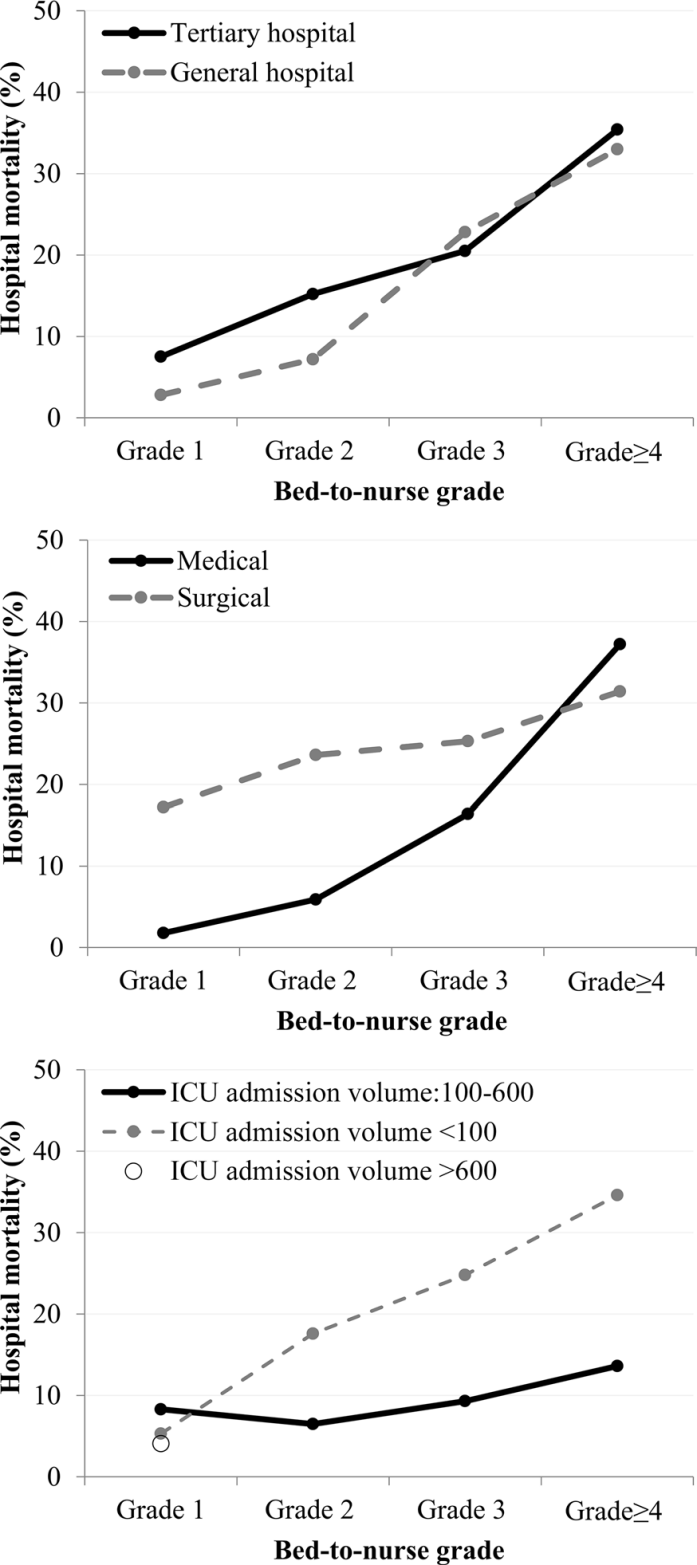
Unadjusted hospital mortality of subgroups according to ICU bed-to-nurse grade in the mechanically ventilated patients

In patients with mechanical ventilation, the positive association between bed-to-nurse ratio and in-hospital mortality was observed regardless of type of hospital and medical/surgical admission department (Fig. 2).

For patients to whom vasopressors were applied but not mechanical ventilation, there was no significant increase in the risk of in-hospital mortality as bed-to-nurse ratios increased (Additional file 2: Table S1).

## Discussion

Our study showed that the adjusted risk of mortality increased as the bed-to-nurse ratio increased in mechanically ventilated patients using population-based nationwide data. The association was consistent regardless of hospital type, or admission department. In the patients without mechanical ventilation, no association between adjusted risk of mortality and the bed-to-nurse ratio was shown. This nationwide study was conducted without bias caused by hospital selection and showed that ICU admission volume was correlated with bed-to-nurse ratio (Additional file 3: Table S2).

There are several possible explanations for the sensitivity of hospital mortality of ventilated patients to bed-to-nurse ratio. Mechanical ventilation requires high workload from the nurse [24, 25], including additional medications (sedatives, neuromuscular blockers), additional procedures (endotracheal intubation, tube maintenance, and chest physiotherapy), and equipment management (ventilator preparation, alarm response, and humidity control). Increased nurse workload is associated with ICU patient mortality [12, 26]. Insufficient physician collaboration, poor nurse–patient communication, increased medical errors, and nosocomial infection are possible causes of the association of high nurse workload and increased mortality [26–29]. Moreover, mechanical ventilation requires intensive monitoring of patients and equipment parameters. Failure of such monitoring may cause life-threatening complications such as pneumothorax or unexpected extubation, which requires prompt recognition and treatment [5, 30]. Shortage of nursing staff could be associated with insufficient supervision and might inhibit early recognition of patients’ changes [31].

For patients on mechanical ventilation, we only found one study about the association of nurse staffing ratio and mortality rate. The study reported no association between number of nurses and mortality in ventilated adult patients [15]. The different results from our study might come from the difference in age of patients or from the different nurse staffing ratios of studying ICUs. Pediatric patients have a short tracheal length, which is a risk for dislodgement, and their developmental immaturity commonly causes poor cooperation with ICU staff [32]. Therefore, more surveillance or workload may be required to care for ventilated children than cooperating adult patients. In the referred adult study, ICU nurses cared for about 2.15 (mean) patients each, and the patient number did not vary across hospitals (standard deviation: 0.37) [15]. However, in our study, the number of patients varied between less than 2.1 (Grade 1) to more than 3.7 (Grade 4 or above). Our study showed that risk of mortality increased in ICU patients as the number of patients increased above 2.1, but we could not evaluate the effect of 1 or less patient cared for by a nurse.

In our study, patients without mechanical ventilation did not show the association between nurse staffing ratio and adjusted mortality rate. According to a recent study, the difference in workload in mechanical ventilation might be a possible explanation for the absence of this association [33]. More importantly, the crude mortality of patients without mechanical ventilation was less than 1.5%, whereas the crude death of ventilated patients ranged from 7.0 to 33.7% (Table 2). We think that the hospital mortality rate of patients without mechanical ventilation might be too low to detect the different consequences of low nurse staffing ratio. However, we cannot conclude that a high nurse staffing ratio is not effective in patients without mechanical ventilation since we do not know the effect of high nurse staffing ratio on outcomes (such as nosocomial infections, readmission rate, and patient satisfaction) other than mortality. We suspect that the different results among studies might be caused by the different proportions of mechanical ventilation or severity of patients in the study units. Severity of patients who use vasopressors likely requires more hemodynamic monitoring and labor, but there was no significant bed-to-nurse effect on in-hospital mortality (Additional file 2: Table S1).

There are many studies about the volume–outcome relationship in critical care [34]. When we adjusted ICU admission volume for mortality, the effect of nurse staffing ratio was offset since the ICU admission volume was correlated with bed-to-nurse ratio (correlation coefficient: 0.714, *p* < 0.01) (Additional file 3: Tables S2, S3). We think the volume–outcome relationship of ICU patients might be partly mediated by unmet standard qualities such as nurse staffing ratio, which might be driven from economies of scale. We could not know how much the nurse staffing ratio mediated the volume–outcome relationship. There could be other mediators of the volume–outcome relationship, such as providers’ experience, education, and technology capacity [34, 35]. However, we could not evaluate all possible partial mediators in this study.

There are some limitations to this study. Using claim data, which is primarily collected for administrative purposes, we could not account for physiologic or laboratory parameters. Although we adjusted patient severity by diagnosis, treatment requirements, and hospital admission factors, there were limitations in adjusting all case-mix severity with the logistic regression model of this study. In addition, the study data were limited to that in the reimbursement system of Korean NHI for ICU staffing ratio. We could not obtain information on the clinical outcomes of a nurse staffing ratio of 1 ventilated patient per nurse compared to 2 ventilated patients per nurse. In this study, we focused on the quantity of nurse staffing; however, the quality of nurse staffing (education course, advanced training) and other ICU team members could be a confounder of mortality. Although we adjusted hospital types that share similar ICU organizational structures or educational programs, the statistical adjustment has limitations.

Despite these limitations, this nationwide scale study provided an overview of the effect of nurse staffing ratio without selection bias of hospital and patient, and highlighted the important effect of mechanical ventilation on the association of nurse staffing ratio and mortality rate.

Our findings also have clinical implications. Even though we know high workload is associated with the poor clinical outcome [33], we could not know the individual patient’s workload before we measure it. However, we could assume the ventilated patients require a high nurse staffing ratio at the time of assigning duty, and nurse allocation should prioritize patients on mechanical ventilation even compared to those using only vasopressors (Additional file 2: Table S1). In addition, our findings have implications related to policymaking. California law limits an ICU nurse to care for a maximum of 2 patients, regardless of the patients’ ventilation state [36]. When a shortage of nurses becomes a concern, it could be considered to refine the regulation to apply to patients on mechanical ventilation.

## Conclusion

As the bed-to-nurse ratio of an ICU increased, the adjusted mortality rate in mechanically ventilated patients increased significantly. Although further studies on ICU volume effects are required, there should be consideration of a policy that limits the number of ventilated patients to 2 or less per nurse in an ICU.

## Supplementary Information

**Additional file 1: Figure S1.** Flowchart of patient selection with inclusion and exclusion.

**Additional file 2: Table S1.** Odds ratio (95% CI) for in-hospital mortality according to ICU grade and combination of mechanical ventilator and vasopressor drugs, 2009 to 2014.

**Additional file 3: Table S2.** Pearson correlation coefficients among hospital variables. Odds ratio for in-hospital Mortality of patients with mechanical ventilation in volume-stratified subgroups.

## Data Availability

The researchers can access on the intranet of Korean Health Insurance Review & Assessment Service through the URL: http://opendata.hira.or.kr/home.do after approval of the request. The researchers can request the same periods, terms and items (claim code) as done in this study. The authors did not have any special access privileges that others would not have.

## Abbreviations

ICU: Intensive care unit
HIRA: Health Insurance Review and Assessment
NHI: National Health Insurance
ATC: Anatomic therapeutic chemical
CI: Confidence interval
