# Supplementary figures and images for: The effect of bed-to-nurse ratio on hospital mortality of critically ill children on mechanical ventilation: a nationwide population-based study
Source: Ann Intensive Care. 2020 Nov 30;10:159. doi: 10.1186/s13613-020-00780-7 (PMC7703514; doi:10.1186/s13613-020-00780-7)

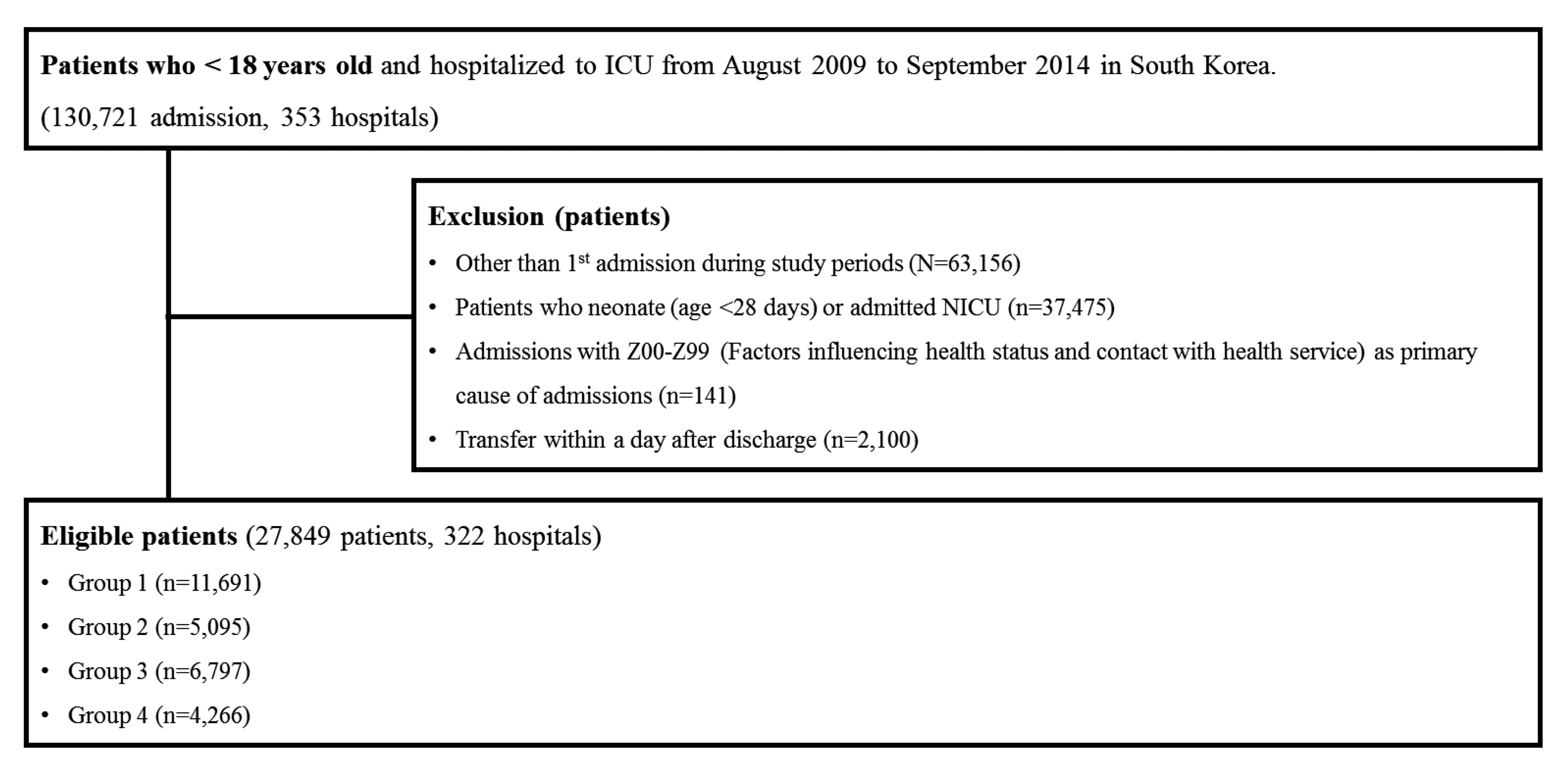

Supplement: Supplementary file 1 — Additional file 1: Figure S1. Flowchart of patient selection with inclusion and exclusion. [file 13613_2020_780_MOESM1_ESM.tif]
