# Supplementary material for: The effect of bed-to-nurse ratio on hospital mortality of critically ill children on mechanical ventilation: a nationwide population-based study
Source: Ann Intensive Care. 2020 Nov 30;10:159. doi: 10.1186/s13613-020-00780-7 (PMC7703514; doi:10.1186/s13613-020-00780-7)
Supplement: Supplementary file 2 — Additional file 2: Table S1. Odds ratio (95% CI) for in-hospital mortality according to ICU grade and combination of mechanical ventilator and vasopressor drugs, 2009 to 2014. [file 13613_2020_780_MOESM2_ESM.docx]

| **Table S1. Odds Ratio (95% CI) for In-Hospital Mortality According to ICU Grade and Combination of Mechanical Ventilator and Vasopressor Drugs, 2009 to 2014** | | | | |
| --- | --- | --- | --- | --- |
| **Bed-to-nurse grade** | MV(-) Vaso(-) | MV(-) Vaso(+) | MV(+) Vaso(-) | MV(+) Vaso(+) |
| **No. death/ No. patients (%)** |  |  |  |  |
| Grade 1 | 48/4798 (1.0) | 21/283 (7.4) | 113/4197 (2.7) | 349/2413 (14.5) |
| Grade 2 | 23/2365 (1.0) | 11/148 (7.4) | 82/1467 (5.6) | 229/1115 (20.5) |
| Grade 3 | 51/4704 (1.1) | 8/173 (4.6) | 123/1116 (11.0) | 286/804 (35.6) |
| Grade 4 or above | 22/3646 (0.6) | 3/104 (2.9) | 74/305 (24.3) | 100/211 (47.4) |
| **Adjusted odds ratio (95% CI)** |  |  |  |  |
| Grade 1 | Reference | Reference | Reference | Reference |
| Grade 2 | 1.18 (0.67-2.03) | 1.45 (0.59-3.59) | 2.41 (1.69-3.43) | 2.41 (1.70-3.43) |
| Grade 3 | 1.39 (0.86-2.25) | 1.33 (0.48-3.73) | 2.89 (2.07-4.05) | 3.82 (2.70-5.40) |
| Grade 4 or above | 0.89 (0.45-1.75) | 0.52 (0.12-2.23) | 7.34 (4.69-11.47) | 4.12 (2.59-6.57) |
| *p-value* | 0.283 | 0.513 | <0.01 | <0.01 |
| MV = mechanical ventilation, Vaso = vasopressor drugs. | | | | |
| Adjusted for age, sex, primary diagnosis, hospital type, medical/surgical admission, interventions for critical care (ECMO and hemodialysis). | | | | |
