# Supplementary material for: The effect of bed-to-nurse ratio on hospital mortality of critically ill children on mechanical ventilation: a nationwide population-based study
Source: Ann Intensive Care. 2020 Nov 30;10:159. doi: 10.1186/s13613-020-00780-7 (PMC7703514; doi:10.1186/s13613-020-00780-7)
Supplement: Supplementary file 3 — Additional file 3: Table S2. Pearson correlation coefficients among hospital variables. Odds ratio for in-hospital Mortality of patients with mechanical ventilation in volume-stratified subgroups. [file 13613_2020_780_MOESM3_ESM.docx]

| **SupplementaRY table 2. Pearson Correlation Coefficients Among Hospital Variables** | | | |
| --- | --- | --- | --- |
| **Hospital Variables** | **ICU bed-to-nurse grade** | **Type of hospital** | **ICU admission volume** ^a^ |
| ICU bed-to-nurse grade |  |  |  |
| Type of hospital | 0.569^*^ |  |  |
| ICU admission volume ^a^ | 0.714^*^ | 0.458^*^ |  |
| Hospital region | 0.568^*^ | 0.547^*^ | 0.488^*^ |
| ^a^ICU admission volume was calculated as the average of annual pediatric admissions to the ICU for 5 years in each hospital. | | | |
| ^*^*p* < 0.001. | | | |

**SUPPLEMENTARY TABLE 3. Odds ratio (95% CI) for In-Hospital Mortality of patients with mechanical ventilation in volume-stratified subgroups**

| **Bed-to-nurse grade** | **No. of patients** | **No. of deaths (%)** | **Model1 OR (95% CI)** | **Model2 OR (95% CI)** |
| --- | --- | --- | --- | --- |
| **ICU admission volume < 100 per year** |  |  |  |  |
| Grade 1 | 226 | 12 (5.3) | Reference | Reference |
| Grade 2 | 1281 | 226 (17.6) | 4.25 (1.52-11.91) | 4.83 (1.46-16.00) |
| Grade 3 | 1489 | 369 (24.8) | 5.50 (1.96-15.42) | 6.65 (2.00-22.12) |
| Grade≥4 | 494 | 171 (34.6) | 8.84 (3.1-25.23) | 12.92 (3.73-44.81) |
| *P-value* |  |  | <0.01 | <0.01 |
| **ICU admission volume 100 - 600 per year** |  |  |  |  |
| Grade 1 | 4420 | 369 (8.3) | Reference | Reference |
| Grade 2 | 1301 | 85 (6.5) | 1.11 (0.54-2.26) | 1.31 (0.52-3.30) |
| Grade 3 | 431 | 40 (9.3) | 1.36 (0.60-3.10) | 1.61 (0.57-4.51) |
| Grade≥4 | 22 | 3 (13.6) | 3.05 (0.65-14.21) | 2.79 (0.47-16.54) |
| P-value |  |  | 0.57 | 0.69 |

*CI*: confidence interval, *OR*: odds ratio, *ICU*: intensive care unit

Model 1 adjusted for age, sex, and primary diagnosis

Model 2 further adjusted for medical/surgical admission, hospital type, interventions for critical care (vasopressor drugs, extracorporeal oxygenation, and hemodialysis)
